# Our New York Letter

**Published:** 1891-02

**Authors:** Wm. L. Russell

**Affiliations:** New York


### Correspondence.

### OUR NEW YORK LETTER.

New York, January 17, 1891.

### THERAPEUTICS OF PNEUMONIA.

A recent number of the *American Journal of Medical Sciences* contains an article on the therapeutics of pneumonia by Dr. A. H. Smith, of this city. It is characterized by the *Medical Record* as the best contribution to the subject which has appeared for years.

Dr. Smith shows that on account of the obstruction to the pulmonary circulation, it is the right heart which bears the greatest burden and which is in danger of being exhausted. On this account he regards the pulse at the wrist as an unreliable guide to the gravity of the condition. The pulmonary valve sound is the proper source of information, and its condition should be carefully noted. If it be markedly accentuated, it indicates that a fairly vigorous right heart is trying to overcome the resistance in the pulmonary circulation. If the accentuation decreases, and there is only moderate dyspnœa, it can be safely felt that the pulmonic resistance is also decreasing. If, however, with the decrease in the accentuation of the pulmonary valve-sound, the respiratory distress increases, the right heart is becoming exhausted. Relief from this condition is to be sought: by regulating the diet in conformity with the diminished power of digestion and sanguification; by the use of medicines which dilate the arteries and promote the transference of blood to them from the veins; by the inhalation of oxygen gas, and by artificial respiration; by placing ligatures about the extremities in order to retain the blood in them, and prevent its return to the heart.

In regard to diet he recommends that the amount of liquid be limited, as it embarrasses digestion and fills the circulatory system. To distribute the blood more evenly throughout the circulation, venesection might be employed. It was better, however, to use the nitrites and nitro-glycerine. Alcohol was also useful as an arterial depressant, as well as a stimulant and a food. Digitalis he did not think desirable. The use of oxygen and of artificial respiration should be resorted to before the condition became critical, and would then be of great benefit.

### CERVICAL ADENITIS IN CHILDREN.

At the last meeting of the Academy the subject of cervical adenitis in children was discussed. Prof. Jacobi introduced the subject by a paper on its etiology, symptomatology, and diagnosis. He questioned the appropriateness of the term gland, as applied to the structures under consideration, and thought that "lymph-bodies" would be preferable. The large number of cells which they contained, and their similarity to embryonic cells, accounted for the frequency of pathological changes in these bodies. The large majority of cases were due to local irritation. In acute cases this was usually easily found; but in the chronic forms it was not always so easily traceable. Normal health, and freedom from abrasions of the integument were the best preventives. Among the most frequent causes might be mentioned diphtheria, and other infectious diseases affecting the mucous membranes; pediculosis and eczema of the scalp; running ears and mastoid abscess. Affections of the nose were also frequent causes, from the most innocent nasal catarrh to ulcers, and the presence of a foreign body. Intestinal parasites usually irritated the mucous membrane, and thus secondarily caused enlarged cervical lymph-bodies. Cracked lips, stomatitis of every form, denuding of the surface by hot food or injury, and dentition, if accompanied by swelling of the gums and irritation, were also to be regarded as causes. Among the rarer causes might be mentioned vaccination, chronic bronchitis, and even pleuro-pneumonia of an upper lobe; in all of these cases, the affection being secondary to inflammation of the axillary and mediastinal lymph-bodies respectively.

The diagnosis was seldom difficult; but the detection of pus was by no means always easy.

The subject was continued by Prof. Wm. H. Thompson, who confined his remarks to the medical treatment. He agreed with Dr. Jacobi regarding the local origin of the condition. He believed, however, that there was a physiological basis for the peculiar proneness of childhood to these inflammations. He had been led to think that the marvellously rapid growth of the brain and nervous system during the first ten years of life demanded such a large supply of blood that the weaker tissues suffered a kind of starvation, in certain children. This was shown in the condition of the hair, the harshness of the skin, catarrhs of the mucous membranes, as eczema and otorrhœa, and phlyctenular conjunctivitis.

The treatment of the condition consisted in the use of proper food, and of cod liver oil, and should be begun as soon as the tendency was discovered. Iodide of potassium had scarcely any effect; iodine, in the form of Lugol's solution, did much better. The best remedy, in his opinion, was, however, calcium chloride. He used the United States officinal solution, in half drachm doses, diluted with syrup of ginger, and administered immediately after meals.

Dr. A. P. Gerster followed Dr. Thomson, with remarks concerning the surgical treatment. He stated that it was the duty of the surgeon to treat the individual as well as the glands. Strict attention should be paid to the causal indications, as treatment based on these was successful in the early stages. In the cases demanding strictly surgical treatment, a division into two classes could be made, namely, acute and chronic. In the acute cases pus was to be suspected whenever there was fever. Œdema of the skin, over the inflamed glands, giving it a pale, shining appearance, could be regarded as almost characteristic of pus under tension in the underlying tissues. Under such conditions, a properly directed incision was the only correct treatment. Continued poulticing was dangerous, and was liable to result in great necrosis of tissue, and even erosions of large blood vessels. It was sometimes difficult to incise so deeply with safety. Danger could be avoided, however, by discarding the knife after division of the skin and fascia. A fine hypodermic needle, attached to a syringe, could then be used to determine the exact locality of the pus. This should afterwards be left *in situ*, as a guide along which to pass a grooved director. The director would then form a guide for a pair of dressing forceps, which, having been introduced, should be opened and withdrawn with the blades apart. The opening could then be enlarged by the little finger, a drainage tube introduced, and a moist dressing applied. In such cases, notably in butchers, fish-mongers, and others who handled dead animal tissues, gland after gland became involved, and the process of incision was interminable. In such cases it was better to remove the whole glandular mass. If a gland containing pus ruptured during the process, the wound should be left open.

Chronic cervical adenitis in children was of pyogenic or tubercular origin. An abscess did not always occur in the pyogenic cases, but whenever pus was detected the best plan of treatment was to aspirate and wash out the cavity. It would refill, but in that case fewer pus corpuscles would be present. When a chronic suppurating adenitis existed it was best to make a free opening and scoop away the remnants of gland tissue. If there were a group of diseased glands, they should be excised. The cause of tubercular adenitis was well known. When there was doubt as to the presence of pus, an aspirating needle should be used to decide. When there was caseation or emulsification of the glands they should be removed soon. If spontaneous perforation had occurred in a number of places, the sinuses should be slit up so as to make one cavity; any bluish, unhealthy looking skin should be removed, as it puckered and made an unsightly scar if allowed to remain; partially broken down glands should be scooped away with a sharp spoon, and the cavity closed or packed. The hemorrhage in such cases was usually profuse, but it was parenchymatous only, and easily controlled. One point should never be lost sight of in aspirating, namely, the possibility of infecting the puncture hole. In one case he had been unfortunate enough to produce even erysipelas. It was, therefore, very important to be certain of the cleanliness of the needle.

Dr. B. F. Curtis remarked that when the tumor was large he was not accustomed to attempt an operation. His reasons for this were, that such operations were prolonged, and accompanied by profuse hemorrhages, neither of which conditions were well borne by children.

I may add that the views advanced by Dr. Curtis are, I believe, similar to those held by most conservative surgeons in New York.

### TUBERCULAR ORIGIN OF LUPUS.

In a late number of the *Medical Record*, Dr. Hyde, the Chicago dermatologist, takes a strong stand against the tubercular origin of lupus. The reasons he gives are as follows:

1. The unimpeachable character of the family history in by far the larger number of cases.
2. The disease in its inception is a disease of childhood, when the habits of the individual are favorable to infection.
3. The sites of predilection are those favorable to infection.
4. The failure to spread by inheritance.
5. The tendency to cutaneous limitation.

In support of his position, he quotes the remarks of Kaposi, at the last International Congress, in which he stated that, in twelve hundred cases observed by him during twenty years of practice, he had never noticed any connection between the disease and tuberculosis.

### KOCH'S REMEDY.

The New York *Medical Journal* speaks the truth regarding the present condition of the relation of Koch's discovery to medical science, in the remark that "the experiments now in progress must be carried on for months before they can lead to any really profitable literature." This is the reason why I have left so little space for relating the progress of events in this connection here. The fact is that there is very little to tell which has not already been told. The excitement which was created by the first announcement of the discovery has disappeared, and, in its place, there is a feeling of calm expectancy, with contentment to wait and see what comes of it. Koch's announcement of the composition of the fluid aroused no special interest, as its nature had been surmised from the beginning. It is being used here now quite extensively, but no positive statements have yet been made. The diagnostic value of the treatment is somewhat questioned, however, as large doses have been required, in some instances, to produce a reaction in undoubtedly tubercular subjects. It may be regarded as proved that lupus can be at least temporarily cured in this way. What it will do for phthisis has not yet been satisfactorily demonstrated.

WM. L. RUSSELL.

